# A Sister Species for the Blue Crab, *Callinectes sapidus*? A Tale Revealed by Mitochondrial DNA

**DOI:** 10.3390/life14091116

**Published:** 2024-09-05

**Authors:** Chiara Locci, Ilenia Azzena, Noemi Pascale, Alessandra Ciccozzi, Ilaria Deplano, Ioannis A. Giantsis, Dimitrios K. Papadopoulos, Athanasios Lattos, Flavio Orrù, Cesare M. Puzzi, Fabio Scarpa, Marco Casu, Daria Sanna

**Affiliations:** 1 Department of Veterinary Medicine, University of Sassari, Via Vienna 2, 07100 Sassari, Italy; c.locci3@phd.uniss.it (C.L.); iazzena@uniss.it (I.A.); npascale@uniss.it (N.P.); marcasu@uniss.it (M.C.); 2 Department of Biomedical Sciences, University of Sassari, Viale San Pietro 43b, 07100 Sassari, Italy; aciccozzi@uniss.it (A.C.); ilariadeplano1@gmail.com (I.D.); fscarpa@uniss.it (F.S.); 3 Department of Chemical, Physical, Mathematical, and Natural Sciences, University of Sassari, Via Vienna 2, 07100 Sassari, Italy; 4 Laboratory of Ichthyology and Fisheries, Faculty of Agriculture, Forestry and Natural Environment, Aristotle University of Thessaloniki, 541 24 Thessaloniki, Greece; igiants@auth.gr (I.A.G.); dkpapado@bio.auth.gr (D.K.P.); lattosad@bio.auth.gr (A.L.); 5 GRAIA Gestione Ricerca Ambientale Ittica Acque, Via Repubblica 1, 21020 Varano Borghi, Italy; flaorru@tiscali.it (F.O.); cesare.puzzi@graia.eu (C.M.P.)

**Keywords:** alien species, MtDNA, Mediterranean invasion, molecular dating, cryptic speciation, sibling species, evolutionary history

## Abstract

The Atlantic blue crab, *Callinectes sapidus*, is acknowledged as one of the worst invasive alien species in the Mediterranean, impacting biodiversity and human activities. Native to the western Atlantic, it has expanded to European coastal waters since the early 1900s. Despite its ecological and commercial importance, genetic research on this species is limited. Here we show a comprehensive investigation of the genetic variation and evolutionary history in *Callinectes sapidus* using 667 mitochondrial COI gene sequences. Our dataset encompasses 36 newly generated sequences from previously understudied Mediterranean sites and 631 from worldwide locations obtained from the GenBank public database. Our findings reveal two distinct, but closely related, genetic groups within the species’ distribution range, suggesting the occurrence of a potential species complex. Furthermore, in the Mediterranean, low levels of genetic variability were observed except for substantial haplotypic differentiation in Turkish samples. This study depicts the global genetic diversity and evolutionary patterns of *Callinectes sapidus*, offering new insights into the taxonomic status of the species.

## 1. Introduction

Non-indigenous species, known as invasive alien species (IAS), pose a significant threat to biodiversity, ecosystem stability, socioeconomic activities, and human health [1,2,3,4]. In this regard, among the widely recognized dangers to biodiversity, the introduction of exotic species stands out as one of the most severe, second only to habitat loss and fragmentation [5]. Over the past decade, the rapid establishment of invasive alien species in marine and coastal ecosystems has emerged as a significant environmental concern, particularly in the Mediterranean region. This latter area is currently acknowledged as one of the most drastically impacted globally, considering both the number of detected alien species and the rate of introduction [6].

In 2006, Streftaris et al. undertook a selection process to identify the one hundred most problematic invasive species in the Mediterranean [2]. In accordance with this list, the Atlantic blue crab, *Callinectes sapidus* Rathbun 1896 (Crustacea, Decapoda, Portunidae), has been designated as one of the most invasive crustacean species in this region [7,8]. Indeed, its presence is noted for its significant impact on biodiversity, fisheries, and aquaculture activities [2], with negative economic and social consequences [9,10]. The blue crab is notorious for causing important damages to fishing nets, primarily through entanglement and cutting [11,12,13,14] and fish mutilations [15]. Due to its aggressiveness [14], this species is known to induce competition with autochthonous species, potentially leading to local species extinctions [15,16].

The blue crab is an opportunistic predator and a scavenger inhabiting the continental shelf in estuaries, lagoons (where it can tolerate freshwater conditions), and near-shore waters. It is found at depths ranging from 0 to 90 m on sandy and muddy substrates, regardless of the presence of vegetation [14,17,18,19]. Additionally, the species is known to thrive in a broad temperature range, from 7 °C to 32 °C, with an optimal temperature of approximately 24 °C in introduced areas [20]. In its native range, however, it can tolerate a wider thermal range, with seawater temperatures reaching up to 40 °C [21] and thermal preferences between 26 °C and 33 °C [22].

*Callinectes sapidus* is a species native to the western Atlantic [23] and holds a dominant position along the eastern coasts of North and South America, including the Gulf of Mexico. In its native range, it represents one of the most important fishery resources for its commercial value and catch volume [24], and it is largely consumed [25,26].

Furthermore, *Callinectes sapidus* is well-suited for colonizing new habitats due to its biology, particularly for its long larval phase and robust swimming capability [14,15,16,27,28,29,30,31,32,33]. Factors such as adult migration patterns, local current and wind patterns influencing larval dispersion, and larval behavior can shape the genetic population structure of the blue crab [15,16].

Since the year 1900, the blue crab has expanded its geographical range to encompass numerous European coastal waters [23]. The presence of *Callinectes sapidus* in Europe was initially reported along the French coasts by Bouvier in 1901 [23,34], followed by reports along the Baltic Sea and the Atlantic coasts of Spain and Portugal [23,35]. The species has also established colonies in Japan since 1975 and Hawaii since 1985 [36], with subsequent expansion to the Black Sea [37] and the whole Mediterranean basin [10,38,39,40]. Historical records suggest its existence in the Aegean Sea as far back as 1935 [23,41,42], but the first confirmed observation in the Mediterranean region was reported in 1949 in Marina di Grado, Italy, in the northern Adriatic Sea. Initial misidentification of specimens as *Neptunus pelagicus* [43] was later corrected to *Callinectes sapidus*, marking the species’ introduction to the region [44]. Subsequent records in Israel, Lebanon, and Egypt further highlighted the spread of the blue crab in the Mediterranean [45,46,47]. In Italy, since the late 1950s, numerous specimens have been collected along the Ligurian [48,49], Tyrrhenian [50,51], Ionian [6,29,52,53] and Adriatic coasts [44,54,55,56,57,58,59,60,61]. Furthermore, in the last decade, the species has exhibited a dynamic expansion in the western parts of the Mediterranean (e.g., [62,63]). Indeed, some specimens were recently recorded along the Sardinian coasts [64,65,66]. Noteworthy, *Callinectes sapidus* has been present for nearly a century in Turkish waters [67,68,69], where its abundance led to the development of a relevant commercial fishery. The significant rise in Turkish fishing activity since the 1980s highlights the potential economic importance of blue crabs for the entire Mediterranean basin [70,71]. However, while blue crab landings are primarily associated with Turkey, they have experienced a significant decline in recent years [28]. Furthermore, although blue crabs have been present in the North Aegean Sea for a similar period [23,41,42], their commercial value in Greece remains lower, as they are considered a secondary fishery product [13,28,72]. Additionally, it is important to note that exploitation of blue crabs also occurs in other Mediterranean regions, including Egypt [47], Tunisia [63], and Italy [57].

Notably, ballast water has been identified as the primary vector for its expansion [23,28,41,42,43,52]. Indeed, initial findings in introduced areas occurred in or near ports where ballast waters were released, suggesting that the species’ introduction is primarily through transport in ballast tanks [23,45,73]. Apart from intentional or accidental introductions via ballast water, potential releases from holding tanks used for live crabs imported for food or the aquarium trade have also been considered [23,74].

However, the rapid and widespread dispersal of *Callinectes sapidus* from introduced areas may also be influenced by long-distance migrations from established populations [23,27]. Adult blue crabs, known for their swimming abilities, can cover substantial distances, with females capable of moving several hundred kilometers [27].

Remarkably, the potential for dispersal of this species not only facilitates the spread of individuals, but also raises concerns about the transmission of pathogens and viral diseases. For instance, the dinoflagellate *Hematodinium* sp. commonly associated with blue crabs can lead to significant mortality in marine crustaceans. The transmission of this parasite from introduced blue crabs to native species can have detrimental effects on indigenous populations [6,72,75,76,77,78]. Another pathogen of concern is the *Callinectes sapidus* reovirus 1 (CsRV1), which was first isolated in blue crabs in the 1970s. While the mechanisms of its transmission and evolutionary dynamics remain largely unknown, CsRV1 has been associated with mortality events in blue crab populations [79,80,81].

Despite the ecological and economic importance of blue crab, genetic research on this species is so far limited. Over the years, several studies have contributed to our understanding concerning the genetic structure and diversity of blue crab populations in both native and introduced ranges. McMillen-Jackson et al. (1994) conducted a pioneering genetic survey of *Callinectes sapidus* across its native range in the United States of America [82]. Place et al. (2005) determined the complete sequence of the blue crab’s mitochondrial genome [83], while Yednock and Neigel (2014) assessed its genetic structure within the Gulf of Mexico, focusing on nuclear protein-coding genes [84]. More recently, Rodrigues et al. (2017) explored the genetic structure of *Callinectes sapidus* populations in Brazil and North America, revealing two distinct lineages in the southern and northern parts of its distribution [85]. Windsor et al. (2019) reported the first analysis of genetic variation across the entire natural range, identifying two lineages within the United States of America to Venezuela area and a third in the region spanning from Brazil to Argentina [86].

Regarding the Mediterranean invasion, Gonzalez-Ortegon et al. (2019) analyzed Atlantic blue crab populations along the Mediterranean coast, discovering two mitochondrial haplotypes (CSWM1 and CSWM2), with varying distribution [68]. Schubart et al. (2023) delved into genetic diversity and gene flow in Mediterranean regions inhabited by *Callinectes sapidus*, confirming three distinct lineages (North American, Caribbean, and South American) in its native range [69]. The same authors also reported that the Mediterranean invasion exhibited low genetic diversity, indicating a recent genetic bottleneck associated with the species’ introduction. The presence of only two frequent haplotypes (CSWM1 and CSWM2) in the Mediterranean suggested a founder effect, emphasizing the potential role of introduction events and environmental factors [69]. The Turkish population, with high genetic diversity, stood out as an exception, likely influenced by a longer divergence time and intentional introductions for enhancement of fisheries [69]. Accordingly, Vecchioni et al. (2022) conducted a focused study on the findings of *Callinectes sapidus* individuals in two rivers of southern Sicily [87]. Subsequently, in 2023, Vella et al. aimed to provide updated records and distribution information regarding *Callinectes sapidus* in Malta and along the eastern coast of Sicily [88].

In such a context, this study aimed to provide first insights into the invasion dynamics of *Callinectes sapidus* across both its native and introduced ranges, with a focus on the Mediterranean areas. To achieve this goal, we assessed the levels of genetic variability within and among *Callinectes sapidus* populations from the Atlantic and Mediterranean coastal regions. To place our Mediterranean samples within a broader geographic context, we analyzed their mitochondrial sequences alongside those reported in the literature to shed new light on phylogeographic patterns. Additionally, species delimitation methods, supported by phylogenetic analysis tools, were employed to enhance our understanding of the evolutionary history of blue crab populations and to evaluate the taxonomy of this species across a large part of its geographical distribution range. Our genetic analyses were based on the analysis of the mitochondrial Cytochrome c Oxidase gene’s subunit I (COI), as, for *Callinectes sapidus*, it is the molecular marker that offers the most extensive sequence data available for comparison in the literature. Our study incorporates over six hundred blue crab sequences representative of various global locations, along with newly obtained sequences from individuals collected in previously underexplored areas of the Mediterranean basin.

This extensive study elucidates the worldwide genetic diversity and evolutionary patterns of *Callinectes sapidus*, providing insights on species’ expansion and adaptation to new environments. Future genetic and morphological surveys, along with pathogen monitoring, are essential for a fully comprehension of speciation events and genetic structuring within the blue crab species. These findings may support effective management strategies in areas where the species is not native and provide valuable hints on the taxonomic status of *Callinectes sapidus*.

## 2. Materials and Methods

### 2.1. Sample Collection

**Figure 1:**
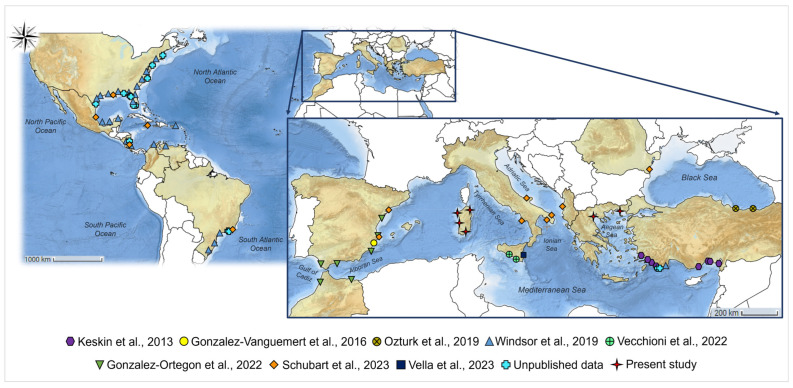
*Callinectes sapidus* sampling plan. The map illustrates the geographical locations where the COI sequences analyzed in the current study were obtained, as well as the locations of sequences from previous research [67,68,69,86,87,88,89,90].

A total of 36 individuals belonging to the *Callinectes sapidus* species was initially identified through morphological observation [44] and collected from various sites along the coasts of the Island of Sardinia and the continental coastal side of Greece in the Aegean Sea (Figure 1 and Supplementary Table S1). Between July 2022 and July 2023, 21 specimens were obtained from four different areas in the northern, central, and southern areas of Sardinia (refer to Figure 1). Among these, 14 individuals were captured in St. Teodoro Lagoon (SS), where the presence of blue crabs is reported for the first time in the present study. Additionally, 2 individuals were collected from Calich lagoon, 1 from the mouth of the Tirso river, and 4 from Santa Gilla lagoon (details provided in Supplementary Table S1). Furthermore, during the month of November 2020, 15 samples were gathered from two locations in the North area of continental Greece: 7 from the Thermaikos Gulf, Chalastra, and 8 from the Rodopi region in Vistonikos Bay. The samples were obtained by collecting a piece of tissue from the walking legs of blue crabs caught by local professional fishermen using trammel nets at the specified locations before being distributed in the local market. No animals were sacrificed in the sampling campaign process, and the sampling method employed was non-invasive. Tissues were stored in absolute ethanol and used for DNA extraction.

This sampling approach received approval from the Ethics Committee (“Organismo Preposto al Benessere e alla Sperimentazione Animale—OPBSA”) of the University of Sassari, under the Protocol number 82235, dated 19 July 2024.

### 2.2. Diagnostic Molecular Analysis

Genomic DNA extraction was performed from a muscle tissue segment using the Macherey-Nagel Nucleo Spin Tissue Kit (MACHEREY-NAGEL GmbH & Co. KG, Düren, Germany) in accordance with the manufacturer’s guidelines. Quantification of DNA solutions was conducted using the Nanodrop™ Lite Spectrophotometer (Thermo Scientific; Waltham, MA, USA), revealing an average yield of approximately 30 ng/μL. A fragment of the mitochondrial Cytochrome c Oxidase gene’s subunit I (COI) was amplified through standard PCR using universal primers [91]. Reactions were executed in a total volume of 25 μL, with an average combination of 10 ng of total genomic DNA, 0.6 μM of each primer, and one PuReTaq Ready-To-Go PCR bead (GE Healthcare, Wauwatosa, WI, USA), encompassing stabilizers, bovine serum albumin (BSA), deoxynucleotide triphosphates, 2.5 units of PuReTaq DNA polymerase, and reaction buffer. Upon reconstitution of a bead to a final volume of 25 μL, the concentrations of each dNTP and MgCl2 were set at 200 μM and 1.5 mM, respectively. PCR cycles were carried out in a GeneAmp PCR System 9700 Thermal Cycler (Applied Biosystems, Waltham, MA, USA) following the specified program: 1 cycle of 4 min at 94 °C, 35 cycles of 30 s at 94 °C, 30 s at 51 °C, and 30 s at 72 °C. Post-treatment involved 10 min at 72 °C and final cooling at 4 °C. Positive (high-quality DNA samples) and negative controls were employed to validate PCR protocol effectiveness and check for possible contaminations. Electrophoresis was conducted on 2% agarose gels, created with 1x TAE buffer (Tris-Acetate-EDTA, pH 8.3), and stained with Gel Red Nucleic Acid Stain (Biotium Inc., Fremont, CA, USA). PCR products were purified with ExoSAP-IT (USB Corporation, Cleveland, OH, USA). Sequencing was performed for both the forward and reverse strands using the same primers employed in the PCR process, and this was carried out by an external sequencing core service (Macrogen Europe, Milan, Italy).

### 2.3. Phylogeographic and Phylogenetic Analysis

Through the Basic Local Alignment Search Tool (BLAST) analysis on the GenBank nucleotide database (www.ncbi.nlm.nih.gov, accessed on 6 May 2024), all newly acquired sequences were confirmed to belong to the species *Callinectes sapidus*, exhibiting a 100% identity match.

Sequence alignment was performed using the Clustal Omega 1.2.4 package [92] (accessible at https://www.ebi.ac.uk/Tools/msa/clustalo/, accessed on 6 May 2024). Manual inspection and editing were carried out using Unipro UGENE v.35 (developed by the Unipro Center for Information Technologies, Novosibirsk, Russia) [93].

A total of 36 blue crab’s sequences spanning the mitochondrial COI region (633 bp) were obtained in the present study (deposited in GenBank under the accession numbers PQ067263–PQ067298). For conducting phylogenetic and phylogeographic analyses within a broader geographic context, these sequences were aligned with 631 COI sequences, reported in literature in previous studies and available on GenBank [67,68,69,86,87,89,90], belonging to blue crabs from the North Atlantic (United States of America—USA and United Mexican States—Mexico)—including the Caribbean Sea (Puerto Rico, Jamaica, Costa Rica, Nicaragua, and Colombia), South Atlantic (Venezuela and Brazil), Western Mediterranean (Spain and Italy), Eastern Mediterranean (Greece and Turkey) and Black Sea (Turkey). Moreover, a subset of 86 sequences was created by combining all Sardinian and Greek sequences acquired in the current study with only those from Turkey, Spain, peninsular Italy, and Sicily (refer to Supplementary Table S2 and Figure S1 for details). This merging aimed to create a wide scenario to depict the phylogeographic and phylogenetic patterns of *Callinectes sapidus* across the Mediterranean and Black Sea regions.

Genetic variation among sequences was evaluated by estimating key parameters, including the number of polymorphic sites (S), number of haplotypes (H), nucleotide diversity (π), and haplotype diversity (h). This analysis was conducted using the software package DnaSP 6.12.03 (developed by Universitat de Barcelona, Barcelona, Spain) [94].

The software JmodelTest 2.1.7 [95] was employed to identify the probabilistic model of sequence evolution that best fit the sequence data, with a maximum likelihood optimized search by the Akaike (AIC) and Bayesian Information Criterion (BIC). The GTR + I + G was suggested by both criteria as the best-fitting model for the whole dataset.

A median-joining network [96] was generated using the software package Network 10.2.0.0 (available at www.fluxus-engineering.com, accessed on 9 May 2024) (Colchester, UK) to deduce genetic relationships among haplotypes and identify potential discrete genetic clusters. Both transitions and transversions were given equal weighting. Given the uncertainty regarding the occurrence of retromutation events, each observed polymorphism was assigned the same weight (10).

To assess the reliability of the entire dataset for taxonomic and phylogenetic investigations, the phylogenetic signal was evaluated using the software TREEPUZZLE 5.3 (Wien, Austria) [97]. A likelihood-mapping analysis of 10,000 random quartets was conducted following Scarpa et al. [98,99]. The Likelihood Map test partitioned the dataset into quartets, representing the smallest set of taxa with multiple unrooted tree topologies [100]. Quartet puzzling operates on groups of four sequences to create a map assessing data reliability for phylogenetic and taxonomic inferences. The key information in the map is the percentage of star-like trees, where a value exceeding 30% indicates that the dataset may not be suitable for analysis due to factors such as noisy data, alignment errors, recombination events, a lack of informative sites, or inadequate taxonomic coverage [98,99].

Phylogenetic relationships were determined through the construction of a Bayesian phylogenetic species tree, generated with the software MrBayes 3.2.7 [101]. The model parameters were set as follows: NST = 6, rates = invgamma, ngammacat = 4. Two independent runs were conducted, each comprising four Metropolis-Coupled MCMC chains (one cold and three heated chains), for a total of 5,000,000 generations. Trees were sampled every 1000 generations, and the initial 25% of the 10,000 sampled trees were discarded as burn-in. Convergence of chains was assessed by ensuring that the Average Standard Deviation of Split Frequencies (ASDSF) approached 0 [101] and the Potential Scale Reduction Factor (PSRF) was around 1 [102], following the methodology outlined by Scarpa et al. [103]. The resulting phylogenetic tree was visualized and edited using FigTree 1.4.0 (accessible at http://tree.bio.ed.ac.uk/software/figtree/, accessed on 15 June 2024).

To validate the taxonomic assessment of the specimens, three species delimitation methods were employed. Firstly, the Automatic Barcode Gap Discovery (ABGD) method [104] was applied. It relies on K2P genetic distances [105] and does not consider the phylogenetic relationships within the dataset. It operates on sequences and identifies the barcode gap as the initial significant gap beyond a specified limit and utilizes it to partition the data [104]. Species evaluation was conducted using the ABGD online tool, accessible at http://www.abi.snv.jussieu.fr/public/abgd/abgdweb.html (accessed on 15 June 2024), with default settings. The appropriate species estimate was chosen following the guidelines of Puillandre et al. [104], employing gene-specific priors for the maximum divergence of intraspecific diversity, corresponding to *p* = 0.001 (refer to Scarpa et al. [106]). The second method used was the nucleotide divergence threshold (NDT), which is based on genetic distances as well. It works on sequences to categorize taxa into taxonomic groups by applying a fixed threshold of 2% established by Hebert et al. (2003) for DNA barcodes [107]. This method employs the pairwise Kimura [105] two-parameter model (K2P) to calculate the genetic distance matrix. The analysis was conducted using a script [98] written in the R statistical environment (available at https://cran.r-project.org, accessed on 15 June 2024). The last method employed for species delimitation was the Poisson Tree Processes (PTP) model and its Bayesian implementation, known as bPTP [108], implemented on the PTP web server (available at http://species.h-its.org/ptp/, accessed on 15 June 2024). This method identifies species boundaries through the phylogenetic species concept (PSC), distinguishing between population and speciation patterns on a given species tree [109]. To conduct this test, the Bayesian species tree constructed using the software MrBayes 3.2.7 [101] was utilized.

### 2.4. Estimation of Divergence Time

The software package Beast 1.10.4 [110] was utilized to estimate the divergence time for the clades identified in the phylogenetic tree. The evolutionary rate of the COI locus in arthropods, which ranges between 0.7% and 2.0% per Myr, with a mean rate of 1.35 per Myr [111,112], was used for calibrating the time tree. The site parameters were configured as follows: Substitution Model = GTR, Bases Frequencies = Estimated, Site Heterogeneity Model = Gamma + Invariant Sites, Number of Gamma Categories = 4, aligning with the evolutionary model chosen by jModeltest. The lognormal uncorrelated relaxed clock model, which assumes independent rates on different branches, was chosen for the molecular clock rate variation, and a “uniform distribution” was selected. The Yule prior process was applied to the tree prior, reflecting the speciation model. Priors for model parameters and statistics were determined to calibrate the time tree based on mutation rates per million years. Operator parameters were set in accordance with the instructions provided in the user manual. Additionally, the utilization of the lognormal uncorrelated relaxed clock model offers insights into the clock-like nature of the data, measured by the ucld.stdev parameter. If the ucld.stdev parameter estimate is close to 0, it indicates that the dataset is relatively clock-like; if the estimated value is significantly greater than 1, it suggests substantial rate heterogeneity among lineages. To achieve an effective sample size (ESS) exceeding 200 for all statistical parameters, a run was conducted with 200,000,000 generations, with a tree sampled at intervals of 20,000 generations, as per the methodology described by Scarpa et al. [103]. The software Tracer 1.6 (©2022 BEAST Developers. All rights reserved.) was employed to examine the resulting log file, aiming to confirm the convergence of parameter values, verify ESS values exceeding 200, and estimate node ages [113]. Tree Annotator (Beast package) and FigTree 1.4.0 were then employed for the drawing and visualization of the time-calibrated tree, following the procedures detailed by Scarpa et al. [103].

## 3. Results

### 3.1. Phylogeographic and Phylogenetic Relationships among Callinectes sapidus Populations from the Whole Distribution Range

A total of 124 polymorphic sites were identified out of 667 sequences analyzed, resulting in 196 distinct COI haplotypes (Table 1). Across the dataset, the highest levels of genetic variation were observed in North American populations, particularly in the Atlantic coast of the United States of America (USA) and in the Mexican coast of the Gulf of Mexico. A general lower rate of genetic diversity was found for Central (Caribbean Sea coasts) and South (Atlantic coasts) America. Within the Mediterranean populations, genetic variation was notably low, except for the Levantine Sea Turkish samples, which displayed a level of variation similar to that of North America.

**Figure 2:**
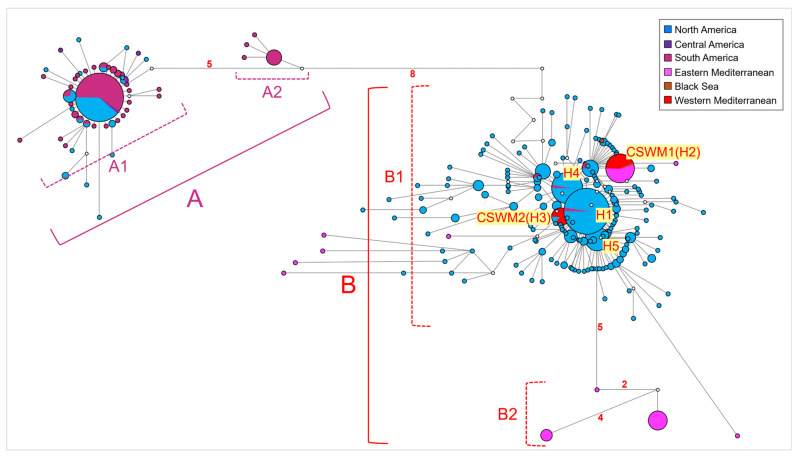
Network (median-joining) analysis performed on the whole dataset of *Callinectes sapidus* mitochondrial COI sequences. Haplogroups A and B and sub-haplogroups A1–A2 and B1–B2 are described in the text. Each circle represents a unique haplotype, with the diameter proportional to its frequency. The number of mutations higher than 1 is reported along the branch length. The small white circles show median vectors, representing intermediate missing or unsampled haplotypes. Network colors indicate the localities of populations investigated, according to the legend.

The network analysis conducted on the entire dataset (Figure 2) revealed a genetic structuring between two primary haplogroups (i.e., groups of haplotypes sharing a common ancestor), designated as groups A and B. Group A included two sub-haplogroups, named A-1 and A-2. Sub-haplogroup A-1 encompassed individuals from South America (Colombia, Venezuela), Central America (Nicaragua and Costa Rica), and North America (USA, Mexico, Puerto Rico, and Jamaica) with a predominant star-like shape. Notably, significant genetic variation was observed in these regions due to the presence of several distinct haplotypes, originating from and surrounding the most frequent haplotype, which was shared by 51.30% of South American (Colombia and Venezuela) individuals and 8.20% of North American (USA, Mexico, Puerto Rico, and Jamaica) individuals. In contrast, sub-haplogroup A-2 was private to 14 Brazilian individuals and diverged for 7-point mutations from A-1. Specifically, it was characterized by one common haplotype and three derived lineages. Group B differed from Group A by 14-point mutations. Notably, 10 of these mutations were silent, meaning they did not result in any amino acid change within COI gene haplotypes. One mutation resulted in missense substitutions, replacing the codon GGC encoding for glycine with two different codons: GTG for valine and AGT for serine. These substitutions can have varying effects on the chemical and physical structure of the protein. The remaining 3 mutations were characterized by degenerate bases, making their precise identification and effect on protein synthesis unattainable. Group B included two sub-haplogroups, B-1 and B-2. Sub-haplogroup B-1 was constituted by samples from North America (USA and Mexico) (likely biased by the higher number of sequences from these areas in the dataset). It also encompassed samples from the Levantine Sea (Turkey), the Aegean Sea (Greece), the Western Mediterranean (Spain, peninsular Italy, Sardinia, and Sicily), the Black Sea (Turkish samples from the Black Sea region), and, to a lesser extent, South America (Venezuela and Brazil). Specifically, sub-haplogroup B-1 included 5 widely distributed haplotypes (named from H1 to H5, in Figure 2), with the most common one (H1) centrally positioned and shared by individuals from North America (USA and Mexico) and a few from South America (Brazil). Surrounding this most common lineage, several derived haplotypes differed by 1- to 3-point mutations, according to a typical star-like shape. One of the five common haplotypes (H2) corresponded to the CSWM1 haplotype, previously reported by Gonzalez-Ortegon et al. (2022) and Schubart et al. (2023) [68,69]. In the current study, CSWM1 was shared by specimens from the Western Mediterranean, Aegean, and Levantine Sea regions, along with a few individuals from the Black Sea coastline. This haplotype was notably prevalent among Mediterranean Turkish specimens (13.9%), 3 individuals from Spain, 52.40% of Sardinian specimens, all Italian peninsular individuals, 2 Sicilian individuals, and all continental Greek samples. Conversely, the CSWM2 haplotype (H3) [68,69] was shared by 2 individuals from Spain, 2 from Sicily, 10 from Sardinia, and 2 from North America (USA). Furthermore, several samples from the southwestern Mediterranean coastline of Turkey exhibited divergent haplotypes that formed the sub-haplogroup B-2. This sub-haplogroup showed the highest genetic affinity with those haplotypes belonging to B-1, which were found in North America (USA). B-2 diverged from these North American haplotypes by 5-point mutations.

Likelihood-mapping analysis, conducted on the entire dataset, reveals a strong phylogenetic signal with a value of white noise of 25.6%, which is below the rejection threshold of 33% [114] (see Supplementary Figure S1).

**Figure 3:**
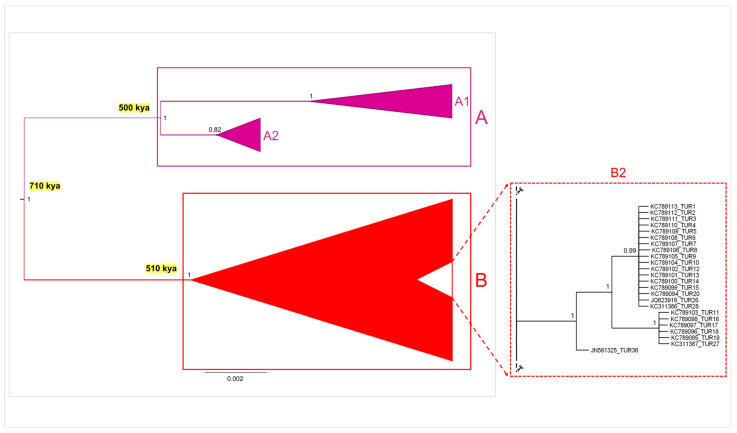
Schematic representation of the Bayesian phylogenetic tree performed on the whole dataset of *Callinectes sapidus* mitochondrial COI sequences (provided at Supplementary Figure S2), integrated with the divergence time estimations at the major nodes (provided at Supplementary Figure S3). Groups A and B and internal clusters A1–A2 and B2 are described in the text. The values at the nodes are represented as posterior probabilities.

Both Bayesian phylogenetic tree and time tree analyses conducted on the complete dataset yielded congruent topologies at the major nodes, fully supported by values of posterior probabilities (pp) of 1. Consequently, to illustrate these findings, a schematic representation (see Figure 3) of the phylogenetic tree generated through MrBayes was crafted, incorporating divergence time information derived from the Beast tree (see Supplementary Figures S2 and S3). Notably, within this representation, emphasis was placed on two distinct monophyletic clades (groups A and B), thereby reinforcing the outcomes of the above-reported genetic clustering analyses (see Figure 2). Groups A and B separated from a common ancestor, which dated back to about 710,000 ya (95% HPD C.I. = 252,000–918,000). Group A, originating approximately 500,000 ya (95% HPD C.I. = 146,000–642,000), included two internal sister clusters (A-1 and A-2). The first, A-1, was predominantly composed of samples from South America (Colombia and Venezuela), with additional representation from Central America (Nicaragua and Costa Rica) and North America (USA, Mexico, Puerto Rico, and Jamaica). The second internal sister cluster, A-2, included most of Brazilian samples. On the other hand, Group B included individuals from North America (USA, Mexico, and Puerto Rico), the Mediterranean region (south-western Turkey, Greece, Spain, peninsular Italy, Sardinia, and Sicily), and the Black Sea region (northern Turkey), along with some remaining samples from South America (Venezuela and Brazil). Group B originated contemporaneously with Group A, dating back to approximately 510,000 ya (95% HPD C.I. = 199,000–696,000). Within Group B, Mediterranean and Black Sea samples were grouped together with most North American and a few South American samples in a large polytomic clade, which included some internal subclusters. Interestingly, a well-supported monophyletic subcluster was identified within this polytomic clade, corresponding to the sub-group B-2 previously identified in the network analysis (Figure 2). This subcluster encompassed the majority of Mediterranean sequences from south-western Turkey. Notably, these Turkish samples displayed an incipient divergence process from the main Mediterranean polytomic clade within Group B. The genetic structuring highlighted in the phylogenetic analyses is consistent with the former clustering analyses, thereby corroborating the outcomes already described for this dataset.

All the species delimitation methods (see Supplementary Table S3) performed on the dataset confirmed the genetic structuring observed between the two main groups of sequences mentioned above. They identified two distinct taxonomic entities, precisely encompassing the sequences structured within the two groups, A and B, already detected in the above reported analyses.

To assess the genetic variation levels within groups A and B, parameters of genetic divergence, such as the number of polymorphic sites (S), number of haplotypes (H), nucleotide diversity (π), and haplotype diversity (h), were calculated also for these two putative taxonomic entities (see Table 2). Group A, consisting of 176 sequences, displayed 50 haplotypes and a lower genetic variation in respect to Group B, which displayed 156 haplotypes out of 491 sequences.

### 3.2. Evolutionary Relationships among Callinectes sapidus Populations, along with Species of the Genus Callinectes

A Bayesian analysis was performed to explore the evolutionary relationships among *Callinectes sapidus* and the other species within the genus *Callinectes*. The analysis generated a phylogenetic tree based on a dataset that included sequences of *Callinectes sapidus* categorized in the two groups (A and B) identified in this study, along with all available sequences belonging to *Callinectes* species from GenBank. Additionally, an outgroup sequence of *Carcinus maenas* was included in the dataset (Supplementary Figure S4).

The results revealed two sequences of *Callinectes bellicosus* that separated from a monophyletic clade containing all the other sequences of the dataset, divided into two main clusters. This monophyletic clade was fully supported at the major nodes by a value of posterior probabilities (pp) of 1 and at the internal nodes by values of posterior probabilities ranging between 0.70 and 1. The main internal cluster of sequences encompassed *Callinectes sapidus* and other *Callinectes* species and included two internal sister subclusters. The first corresponds to a monophyletic clade encompassing sequences of *Callinectes danae*, *Callinectes similis*, *Callinectes arcuatus*, *Callinectes ornatus*, *Callinectes exasperatus*, and *Callinectes pallidus*. The second corresponded to a large monophyletic clade grouping sequences of *Callinectes sapidus* along with sequences belonging to other species of the genus *Callinectes*. Within this clade, *Callinectes larvatus* and *Callinectes marginatus* branched off separately from *Callinectes sapidus* species, which clustered together with *Callinectes bocourti*, *Callinectes rathbunae*, *Callinectes amnicola*, and *Callinectes toxotes*.

### 3.3. Genetic Variation of Callinectes sapidus within Its Mediterranean and Black Sea Ranges of Distribution

**Figure 4:**
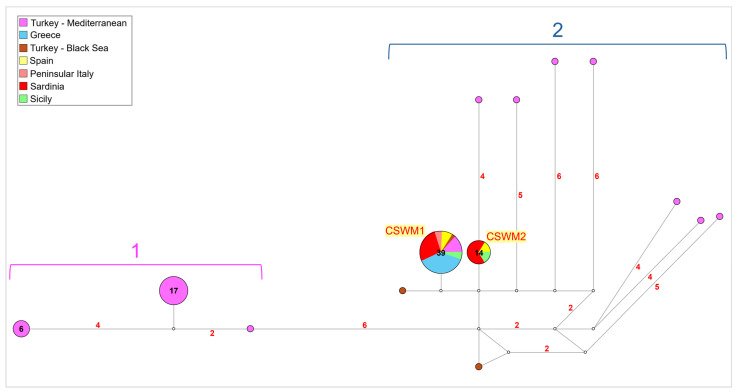
Network (median-joining) analysis performed on the Mediterranean and Black Sea subsets of mitochondrial COI sequences analyzed for *Callinectes sapidus*. Groups 1 and 2 are described in the text. Each circle represents a unique haplotype, with the diameter proportional to its frequency. The number of mutations higher than 1 is reported along the branch length. The small white circles show median vectors, representing intermediate missing or unsampled haplotypes. Network colors indicate the localities of populations investigated, according to the legend. The number of individuals sharing the same haplotype, with a frequency greater than 1, is displayed within the circle.

The network analysis conducted on the subset of samples from the Mediterranean and Black Sea regions revealed significant genetic structuring between two distinct groups, Group 1 and Group 2, both exhibiting notable founder effects as suggested by the star-like shapes represented in the graphic (Figure 4). Group 1 consisted of 24 Turkish individuals, mainly from the south-western Mediterranean area of the country, showing a divergence of 6-point mutations from Group 2. In contrast, Group 2 comprised two main common haplotypes. The first haplotype, corresponding to the already reported CSWM1 [68,69], was shared by 39 individuals, mainly from Sardinia and continental Greece, with some representation from Spain, peninsular Italy, Sicily, and the Levantine Mediterranean coast of Turkey. The second haplotype, corresponding to the already reported CSWM2 [68,69], was found in 14 individuals, mainly from Sardinia and a few from Spain and Sicily as well. Additionally, within Group 2, several private haplotypes, corresponding to the Levantine and Black seas Turkish sequences, diverged by 2- to 10-point mutations from the main Mediterranean haplotypes.

## 4. Discussion

The current study represents an extensive molecular investigation conducted on the blue crab (*Callinectes sapidus*) populations, encompassing a dataset sourced from the Atlantic coasts of North, Central and South America, and various Mediterranean and Black Sea regions hitherto underexplored. Notably, this study examined the distribution of genetic variability within the Mediterranean Sea, acquiring sequences from continental Greece and Sardinia in greater abundance compared to extant sequences from the Mediterranean already reported in literature [67,68,69,86,87,89,90].

Combined phylogenetic and species delimitation analyses led to the most striking result of the ongoing research, which is the appraisal of two closely related, but sharply distinct, genetic groups within a large part of the distribution range of *Callinectes sapidus*. Such a finding suggests the existence of two possible sister species, indicating the occurrence of a species complex for *Callinectes sapidus*.

Accordingly, clustering analyses have highlighted the presence of two strongly differentiated genetic groups, separating most of the South Atlantic American blue crab populations from those in the North Atlantic, Mediterranean, and Black Sea. Phylogenetic analyses have further corroborated this finding by identifying two distinct *Callinectes sapidus* mitochondrial lineages. Notably, Schubart et al. (2023) have already recognized genetic disparities between the North and South Atlantic blue crab populations through mitochondrial COI gene analyses [69]. This previous study reported restricted gene flow within the two regions, likely due to repeated isolation in water bodies of different sizes. Similarly, Rodrigues et al. (2017) reported two distinct lineages in the northern and southern parts of blue crab American distribution [85], while Windsor et al. (2019) identified two lineages within the United States of America to Venezuela area and a third lineage from Brazil to Argentina [86], both using the mitochondrial COI gene as molecular marker. It is worth mentioning that the study by Rodrigues et al. [85] employed a portion of the COI gene that did not align with our dataset, which is why their sequences were not included in our current analysis.

Interestingly, phylogenetic analysis gave us the possibility to evidence the contemporary divergence of the two *Callinectes sapidus* putative sister species on the North and South Atlantic American coasts, which originated around 500,000 years ago. We hypothesized that these two sister taxa were raised from a common ancestor that likely spread along the entire American Atlantic coastline after its early origin about 700,000 years ago. Interestingly, this evolutionary period coincided with the Early-Middle Pleistocene Transition (EMPT), a significant climatic shift occurring around 1.2 million years ago [115]. The EMPT, previously known as the Mid-Pleistocene Transition, was characterized by intensified climate oscillations with 100,000-year periodicity, notable asymmetry in global ice volume cycles, and a reduction in the duration of interglacial periods starting from 1.4 million years ago [115]. This transitional phase persisted until about 420,000 years ago, after which high-amplitude interglacial periods occurred. Marine records from this period revealed high-frequency climate variability influenced by factors such as precessional forcing, changes in ice sheet dynamics, temperature variations, and alterations in oceanic currents [115]. Notably, during the interval from 712,000 to 676,000 years ago, an interglacial period occurred, with an extended deglaciation and sustained interglacial conditions spanning across two summer insolation peaks [115].

Furthermore, the patterns of genetic diversity between the two taxonomic entities we observed in *Callinectes sapidus* can be explained by the environmental conditions during the Marine Isotope Stage 13 (MIS 13) period, approximately from 533,000 to 478,000 years ago. During MIS 13, the Northern Hemisphere experienced a robust summer monsoon and warmer interglacial conditions, marked by rising temperatures and declining ice volumes [115,116]. These environmental changes likely had a significant impact on the migration patterns and adaptation of various tropical marine organisms, including invertebrates, temperate fish, and marine mammals [117].

For what the blue crab is concerned, in the southern regions of American coastline, the observed lower genetic diversity compared to the Northern Atlantic area can be attributed to a positive reinforcement of the two distinct evolutionary forces: genetic drift, as the founder effect, and natural selection, as the selective sweep. Indeed, the warmer and more favorable environmental conditions during MIS 13 may have led to the selection and the spreading of specific, adaptive haplotypes in the blue crab southern populations. This rapid adaptation and massive expansion of the adaptive haplotypes resulted in a reduction of the overall genetic diversity in the southern region, as the population became dominated by positive selected lineages. As the adaptive founder haplotypes became established in the southern populations, the evolutionary rate likely gradually decreased over time. In this context, since the blue crab populations had already acclimated to the prevailing environmental conditions, further adaptation was likely unnecessary. Additionally, a continuous and effective gene flow due to larval dispersal across the western South Atlantic, as reported by Lacerda et al. (2016) in modern populations, could have homogenized genetic variation within past populations, further reducing diversity over time [118].

In such a context, our study has evidenced an incipient, yet statistically relevant, mitochondrial genetic structuring between the two putative sister species of the blue crab. Notably, despite these differences, the two species maintain a considerable level of genetic similarity among each other. Furthermore, our results highlighted a relevant genetic divergence between the two putative blue crab sister species that are representative of the *Callinectes sapidus* species complex and the other species belonging to the genus *Callinectes*. It is important to take into consideration that the glacial stage preceding MIS 13 may have affected connectivity between different marine organisms in the North and South Atlantic. The lowering of sea levels during this period could have created, or increased, the geographic barriers, potentially prompting species diversification [115,117].

Despite the emerging genetic structuring between the North-Central and South American populations of *Callinectes sapidus*, significant morphological distinctions have not been reported to date. This suggests that the mitochondrial incipient speciation events may be related to environmental conditions, but nucleotide diversification has possibly not yet progressed sufficiently to yield detectable morphological differences between the two putative sister species. To fully understand the extent of genetic divergence between North-Central and South American populations, as well as the potential presence of underlying morphological variations that may have been previously undetected, further comprehensive genetic and morphological investigations are essential. Future studies should focus on analyzing both nuclear and mitochondrial markers in *Callinectes sapidus* individuals collected from the entire Atlantic coastline of the Americas.

The abovementioned scenario, which depicts the presence of two genetically divergent putative sister species for *Callinectes sapidus* inhabiting North-Central and South America coastlines, is strongly and independently further corroborated by a study conducted by our research group (Scarpa et al., submitted), which aims to perform a phylodynamic genome-based reconstruction incorporating all *Callinectes sapidus* Reovirus 1 (CsRV1) genomes and segments available in the NCBI virus database. Indeed, it should be taken into consideration that pathogen dispersal is typically influenced by host movement, providing crucial insights into host migration and population connectivity [81,119]. In this context, preliminary results of our phylodynamic analysis on the CsRV1 highlighted a genetic structuring between the viral strains from North Atlantic and South Atlantic American coastlines, which is consistent with the significant genetic divergence evidenced in the present study among the blue crab populations from these two oceanic waterbodies.

Moving our discussion to the Mediterranean and Black Seas, the haplotypes reported for this area in the present study belong to the *Callinectes sapidus* taxonomical entity that we found to be exclusive of North America Atlantic coastline. This observation suggests that the demographic expansion of *Callinectes sapidus* in the Mediterranean might have been facilitated by the introduction of blue crabs into the basin mainly through ballast waters [23,28,41,42,43,52].

In this context, the predominant Mediterranean and Black Sea haplotypes correspond to, or closely resemble, those distributed in North America. Consistently with Schubart et al. (2023), the genetic landscape evidenced for this area in the present study is characterized by the presence of the two most frequent reported mitochondrial lineages (i.e., the so-called CSWM1 and CSWM2) (see [68,69] for details), along with several closely derived haplotypes suggesting that the occurrence of a marked founder effect may have affected the spreading of blue crabs in the Mediterranean and Black Sea areas [69].

The present study has revealed that the CSWM1 haplotype is predominant in the Mediterranean basin, corroborating the findings of previous research. Specifically, González-Ortegón et al. (2022) reported this haplotype to be prevalent in the Alboran Sea and the Gulf of Cadiz [68], and Schubart et al. (2023) found it to be also widespread in the southern Adriatic and Black Sea [69]. Furthermore, the CSWM1 haplotype was also detected in Sicily [87]. Importantly, the current study has provided evidence of the presence of CSWM1 also in geographic areas that were previously poorly investigated, such as continental Greece and the island of Sardinia. Additionally, in the present study, the CSWM2 haplotype has been found to be predominant along the western Mediterranean coasts of Spain, as previously reported by González-Ortegón et al. (2022) and Schubart et al. (2023) [68,69]. We also detected for the first time this haplotype on the island of Sardinia, and, as it was previously reported [87], on the island of Sicily. These findings contribute to a more thorough understanding of the genetic diversity and distribution of *Callinectes sapidus* within the Mediterranean and Black Sea areas. To further expand this knowledge, future research efforts should focus on collecting data from additional locations in these areas, which would deepen our comprehension of the distribution patterns of the CSWM1, CSWM2, and derivate haplotypes across this large geographic region.

Interestingly, a significant genetic differentiation has been evidenced in the present study between individuals originating from Turkey and those from other Mediterranean areas, despite the relatively close geographic proximity to some other Mediterranean regions, such as the North Aegean (see Figure 4). Particularly, Turkish individuals from the Levantine Mediterranean south-western coastline of the country displayed the highest levels of mitochondrial divergence from the other Mediterranean populations. The high level of genetic divergence in Turkish blue crabs, with the occurrence of at least two large groups of mitochondrial lineages, likely stems from their early arrival in the region around a century ago [67,68,69]. This predates the much more recent, massive appearance of the species in the western Mediterranean. Although blue crab may have faced difficulties establishing in other parts of the Mediterranean after its initial introductions, the species could have experienced a rapid adaptation in Turkey, where local environmental conditions might have been conducive to its survival and genetic differentiation since its first arrival.

In particular, first sightings of the blue crab in Thermaikos Gulf date back to 1935 [23,41,42], suggesting that vigorous commercial maritime interactions between the Aegean coast of Greece and Mediterranean Turkey might have accelerated the spread of *Callinectes sapidus* in this latter area earlier than in other Mediterranean regions [120].

Consequently, the high level of internal genetic variation observed in the present study among Turkish blue crabs may be the consequence of multiple introductions over the past century that produced an overlapping of different haplogroups (distinct groups of similar haplotypes with different common ancestors) in the country. However, an alternative scenario should also be considered to explain the occurrence of multiple, and divergent, mitochondrial lineages in Turkey. Considering that commercial shipping trade is less intense in southern Turkey compared to other areas of the country and also considering that fisheries and blue crab harvesting represent an important economic national resource, the genetic pattern reported in this study for the Turkish Levantine Mediterranean region may be the result of intensive human-mediated activities that acted as artificial evolutionary forces driving divergence from other Mediterranean areas.

Conversely, the limited genetic variability and the marked signs of founder effects detected for the other Mediterranean regions here analyzed align with a species newly established in the area, predominantly comprising descendants of recently introduced founder individuals. In this context, based on our data, the early reports of blue crabs in the western Mediterranean, dating back to the mid-20th century (i.a., [6,29,48,49,50,51,52,53,62,63,64,65,66]), might have been followed by a rapid disappearance of the species due to incompatibility with the environmental conditions that, at that time, were not suitable with the expansion of the species in those geographical areas.

On the other hand, the present general mitochondrial homogeneity observed in the modern Mediterranean blue crab populations is likely attributable to the species’ high larval dispersal capability [15,69]. This has resulted in the predominance of common haplotypes, likely adapted to environmental conditions, across the region. Moreover, marine currents may have further facilitated the gene flow among blue crab populations, thus contributing to the reduced genetic variability observed among distant Mediterranean sites for this species [121,122]. Additionally, the genetic similarities observed between populations from the Black Sea and the Mediterranean suggest a possible pathway for the introduction of *Callinectes sapidus* into the Black Sea from the northern Aegean Sea, likely via the Gulf of Saros, where sightings have been recorded since 1935 [23,67,69]. Therefore, our results confirm what was proposed by Ozturk et al. in 2020, which hypothesized that individuals of *Callinectes sapidus* migrated from the Saros Bay to establish a thriving population in the Black Sea. Again, in this case, the high dispersal capability of larvae and notable adults’ swimming capacity may also account for these results [67,69].

Interestingly, the exclusive haplotypes that are private to the Mediterranean Levantine Sea Turkish population were not found in the modern North American population, which is the likely source of blue crab introductions to Turkey a century ago. This finding could be attributed to the effect of evolutionary forces, such as genetic drift or selective sweep, acting on the first founding population, which was introduced to Turkey in the early 1900s.

This phenomenon is consistent with the fundamentals of adaptive evolution, according to which the molecular lineages of introduced populations can be rare, or became absent, in the source population but prevalent among individuals in the newly colonized region [123]. This is likely due to founder events or natural selection favoring the spread of rare, potentially adaptative alleles, thereby enhancing the species’ adaptability to the new environment. This scenario is supported by similar findings in other invasive Mediterranean and European species, such as the teleost *Fistularia commersonii* [124] and the crustacean *Procambarus virginalis* [125], where recently introduced populations lacked mitochondrial lineages present in the source populations. However, another plausible explanation for the existence of private haplotypes in the Turkish population, which are nowadays absent in North America, is the potential extinction of these lineages in their native range during the last century. This extinction could have occurred over time, possibly due to natural cycles of demographic fluctuations in the North American blue crab populations. Consequently, these haplotypes have become endemic and private to the Turkish population only.

## 5. Conclusions

Our study presents a wide geographical examination of the genetic diversity of the blue crab (*Callinectes sapidus*) populations, encompassing a large mitochondrial dataset from the Americas and previously under investigated European and Mediterranean regions. This research reveals significant insights into the species’ evolutionary history and its phylogeography, including the identification of two distinct genetic groups within the blue crab populations, suggesting the potential existence of a never-reported sister species predominantly spanning in southern America. This study also sheds light on the introduction pathways and genetic adaptation of *Callinectes sapidus* in new environments of the Mediterranean and Black Sea. Furthermore, our findings highlight the role of natural selection and genetic drift in shaping the genetic diversity of introduced populations.

In conclusion, this research provides valuable insights into the genetic diversity, evolutionary history, and phylogeographic patterns of *Callinectes sapidus* with important implications for the understanding of the species’ expansion across new environments. Future genetic and morphological investigations, along with studies on reproductive biological traits, are essential to deepen our comprehension of the evolutionary processes and taxonomical status of *Callinectes sapidus*. These studies are fundamental for corroborating the occurrence of speciation events within the species complex of *Callinectes sapidus* and for determining whether the two putative sister species could be considered as cryptic or sibling species. Additionally, monitoring pathogens, particularly CsRV1, would be crucial for better depicting the genetic structuring among blue crab populations.

## Figures and Tables

**Table 1:** Sample sizes and genetic diversity estimates obtained for the mitochondrial COI region analyzed for *Callinectes sapidus* individuals. N: sample size; S: number of polymorphic sites; H: number of haplotypes; h: haplotype diversity; π: nucleotide diversity. Sites with gaps were not considered.

Geographic Region	N	S	H	h	π
United States of America	397	100	146	0.922	0.00766
Puerto Rico	22	34	10	0.749	0.00600
Jamaica	1	0	0	0.000	0.00000
United Mexican States	43	38	22	0.844	0.01777
**North America**	463	108	162	0.931	0.01249
Nicaragua	4	2	3	0.833	0.00184
Costa Rica	1	0	0	0.000	0.00000
**Central America**	5	4	4	0.900	0.00284
Venezuela	90	45	30	0.614	0.00216
Colombia	4	2	3	0.833	0.00158
Brazil	19	22	7	0.667	0.01168
**South America**	113	53	39	0.729	0.00712
**Turkey—Black Sea**	3	5	3	1.000	0.00527
Greece	15	0	1	0.000	0.00000
Turkey—Levantine Sea	36	46	11	0.744	0.01317
**Eastern Mediterranean**	51	45	11	0.733	0.01289
Spain	5	3	2	0.600	0.00292
Peninsular Italy	2	0	1	0.000	0.00000
Sardinia	21	3	2	0.524	0.00248
Sicily	4	3	2	0.500	0.00237
**Western Mediterranean**	32	3	2	0.508	0.00247
**Whole Mediterranean**	83	45	12	0.725	0.01103
**Whole dataset**	667	124	196	0.937	0.01793

**Table 2:** Sample sizes and genetic diversity estimated for the whole dataset of *Callinectes sapidus* mitochondrial COI sequences categorized into the two groups A and B retrieved by genetic clustering and phylogenetic analyses. N: sample size; S: number of polymorphic sites; H: number of haplotypes; h: haplotype diversity; π: nucleotide diversity. Sites with gaps were not considered.

Group	N	S	H	h	π
A	176	60	50	0.685	0.00349
B	491	110	156	0.933	0.00699

## Data Availability

Sequences obtained in the present study for the mitochondrial COI gene were deposited in the GenBank database under the accession numbers PQ067263–PQ067298.

## Supplementary Materials

The following supporting information can be downloaded at: https://www.mdpi.com/article/10.3390/life14091116/s1. Figure S1: Phylogenetic signal performed on the whole dataset of *Callinectes sapidus* mitochondrial COI sequences. The likelihood-mapping method partitions the area of the equilateral triangle into seven regions. The three trapezoids at the corners represent the areas supporting strictly bifurcating trees, that is, the presence of a tree-like phylogenetic signal. The three rectangles on the sides represent regions where the decision between two trees is not obvious. The center of the triangle represents sets of points P (posterior probabilities of the unrooted trees) where all three trees are equally supported (Scarpa et al., 2019) [98]. Figure S2: Bayesian phylogenetic tree obtained by the software MrBayes 3.2.7 and performed on the whole dataset of *Callinectes sapidus* mitochondrial COI sequences. The values at the nodes are represented as posterior probabilities. The sequences obtained in the present study are reported in red (Sardinia) and light blue (Greece). Figure S3: Ultrametric tree obtained by the software Beast 1.10.4 and performed on the whole dataset of *Callinectes sapidus* mitochondrial COI sequences. Divergence times among taxa are shown. The sequences obtained in the present study are reported in red (Sardinia) and light blue (Greece). Figure S4: Bayesian phylogenetic tree obtained by the software MrBayes 3.2.7 and performed, including the mitochondrial COI gene sequences of *Callinectes sapidus* along with sequences from other *Callinectes* species sourced from GenBank. The values at the nodes are represented as posterior probabilities. Table S1: *Callinectes sapidus* specimens collected in the present study. The table reports the samples’ code, GenBank accession numbers, sampling locations, sampling date, and species. Table S2: COI dataset. The table shows *Callinectes sapidus* COI sequences used in the present study, taken from the GenBank database. Sample code, GenBank accession numbers, collection locations and period, species, and references are reported. Table S3: Species delimitation results. The table shows the outcomes from the species delimitation methods applied to the whole *Callinectes sapidus* mitochondrial COI dataset. All three methods employed (ABGD, NDT, and PTP) produced identical results. Table S4: *Callinectes* genus COI dataset. The table shows the sample code and the GenBank accession numbers of species belonging to the *Callinectes* genus, taken from the GenBank database.
