# Supplementary material for: A Sister Species for the Blue Crab, Callinectes sapidus? A Tale Revealed by Mitochondrial DNA
Source: Life (Basel). 2024 Sep 5;14(9):1116. doi: 10.3390/life14091116 (PMC11433012; doi:10.3390/life14091116)

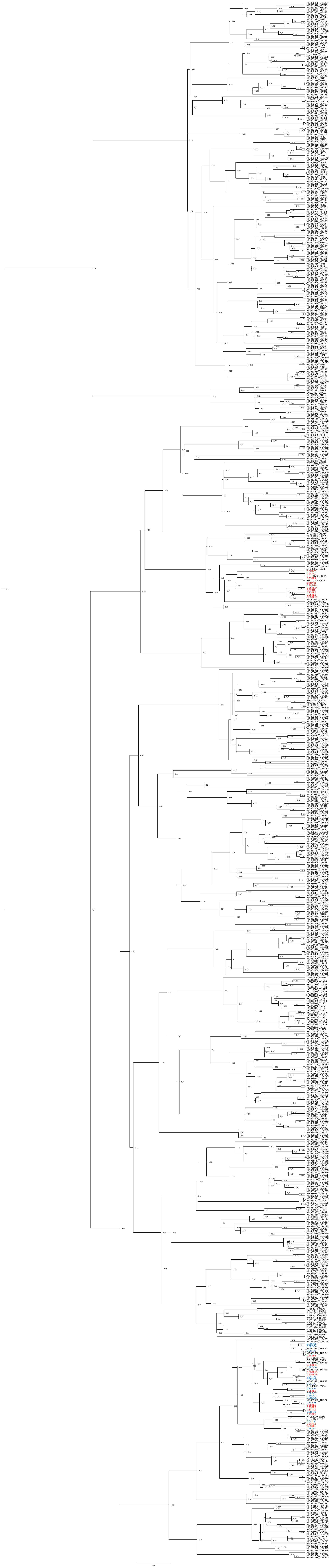

Supplement: Supplementary file 1 [file Figure S3.pdf › Figure S3.pdf]
