# Supplementary material for: A Sister Species for the Blue Crab, Callinectes sapidus? A Tale Revealed by Mitochondrial DNA
Source: Life (Basel). 2024 Sep 5;14(9):1116. doi: 10.3390/life14091116 (PMC11433012; doi:10.3390/life14091116)
Supplement: Supplementary file 1 [file Figure S4.pdf › Figure S4.pdf]

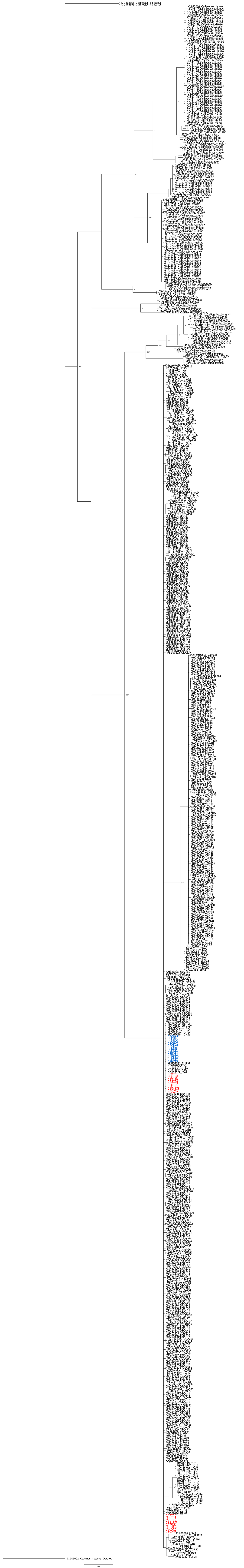

MG462556_Callinectes_bellicosus
MG462555_Callinectes_bellicosus
OQ108526_ESP2
ON248059_ESP5
CSSTE2
CSSTE4
CSSTE7
CSSTE11
CSSTE14
CSTIR1
CSCAG1
CSCAG2
CSCAG3
CSCAG4
JQ306002_Carcinus_maenas_Outgroup
0.02
